# A safe and simpler diagnosis of gastric schwannomas

**DOI:** 10.1002/ccr3.215

**Published:** 2015-02-02

**Authors:** Rambod Charepoo, Arun P Palanisamy, Jayakara Shetty, Scott H Kurtzman

**Affiliations:** 1 Department of Surgery, Medical University of South CarolinaCharleston, South Carolina, 29425; 2 Department of Surgery, Waterbury HospitalWaterbury, Connecticut, 06708

**Keywords:** Diagnosis, diagnostic biopsies, gastric schwannomas, MRI, percutaneous, seeding

## Abstract

Although gastric schwannomas usually are nonmalignant, these tumors can undergo malignant transformation. For diagnosis, endoluminal routes are believed to decrease the chance of cancerous cell dissemination. We present a case where a percutaneous route was utilized with supporting evidence for the safe use of this method for diagnosis.

## Introduction

Although schwannomas are generally considered to be benign tumors, they can potentially undergo malignant transformation 1. Hence, precautionary measures are undertaken to ensure prevention of cancer cell seeding during diagnostic biopsies. It has been a long-held belief that the safest way to approach these tumors for diagnostic purposes is through endoluminal routes, decreasing the chance of dissemination of cancerous cells 2. There is now mounting evidence suggesting that biopsies for cancers in general can be done in a safe and controlled fashion with small gauge needles without an increase in tumor seeding or cancer dissemination 3,4. We present a case of a gastric schwannoma (GS) in which a percutaneous route was undertaken, with supporting evidence of the safe use of this method for diagnosis.

## Case Presentation

**Figure 1:**
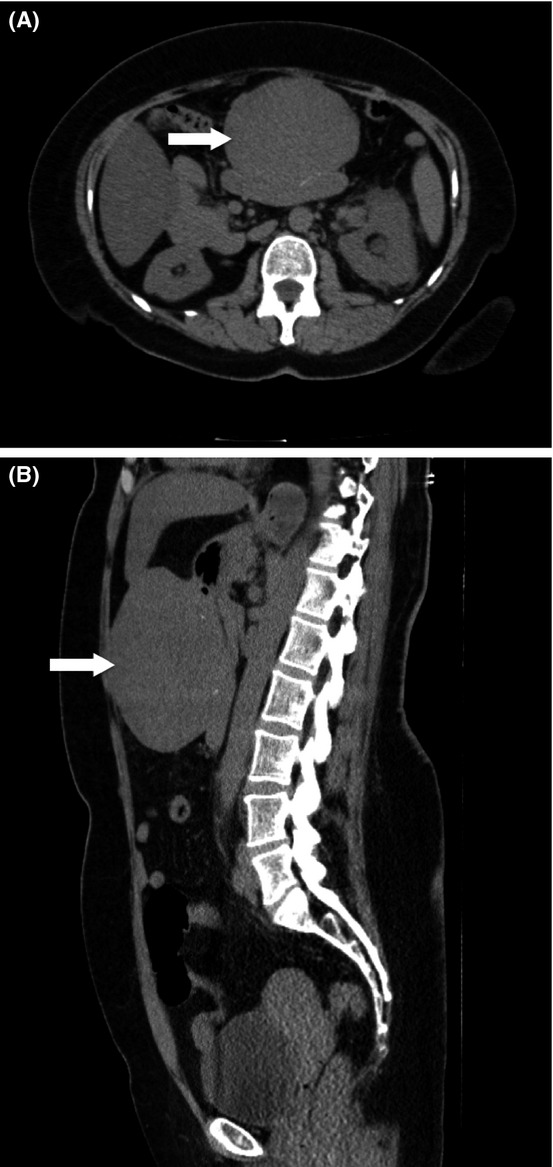
CT scan of patient's abdomen shows an incidentally discovered homogeneous, solid mass that displaced the stomach posteriorly.

Patient was a 56-year-old white female. She was initially seen in the hospital emergency department for flank pain suggestive of nephrolithiasis. The patient had no abdominal symptoms or complaints except her flank pain. CT scan of her abdomen done at that time confirmed a small renal calculus in the distal portion of her left ureter with moderate hydronephrosis and hydroureter. There was an incidentally discovered homogeneous, solid mass that displaced the stomach posteriorly (Fig.1A). The mass appeared to originate from the anterior stomach wall, and measured 10 × 8 cm in its greatest dimensions (Fig.1B). Several lymph nodes were also identified in the gastrohepatic ligament. The radiologic findings indicated a gastrointestinal stromal tumor (GIST). A directed abdominal examination revealed a large nontender abdominal mass in the midepigastrum.

**Figure 2:**
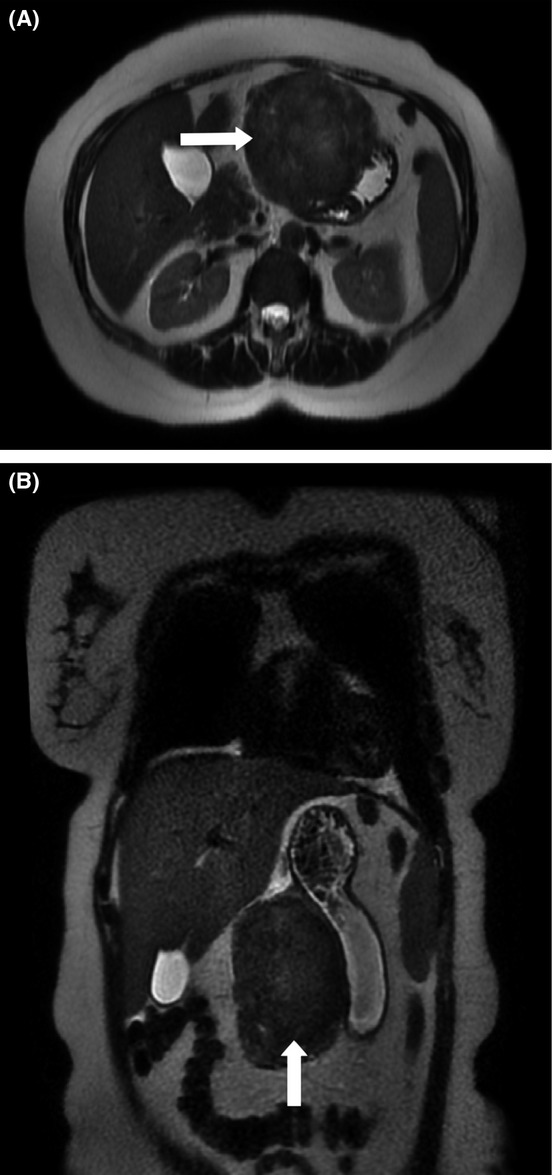
Abdominal MRI confirmed a large discretely marginated mass centered in the region of anterior stomach wall and the lesser curvature.

Subsequently, abdominal MRI confirmed a large mass, discretely marginated, in the anterior stomach wall and the lesser curvature. The MRI findings were consistent with GS as described by Karabulut et al. (Fig.2A and B) 5.

The patient's laboratory findings were normal. Although she was asymptomatic from the mass, she was sent for an upper endoscopy and a possible biopsy. Upper endoscopy failed to show any intraluminal pathology. Due to the benign attributes and fibrous appearance of the mass on imaging studies, it was felt a percutaneous needle biopsy would be safe and feasible. Therefore, an US-guided biopsy was suggested. Three biopsies taken from the extrinsic mass revealed a variable spindle-cell lesion composed of plump and wavy cells with tapered ends and inconspicuous cytoplasm, predominantly in short fascicles, with focal areas of nuclear palisading, hyalinized vessels, and admixed chronic inflammation. There was no significant nuclear atypia or mitotic activity, which would suggest malignancy. The immunohistochemical profile showed positive S100 staining and was negative for cKIT, SMA, Desmin, Caldesmon, and CD34 staining. Morphologic and immunophenotypic features of the specimen were identical to that of a peripheral nerve sheath tumor, most consistent with a schwannoma.

## Outcome

Based on the findings, resection of the mass was recommended to the patient. At laparotomy, the abdominal mass was adherent to the anterior wall of the mid-stomach. It was pedunculated and easy to manipulate during the procedure. There was no invasion of any other organs and the base was noted to be thin and elongated. A stapling device was used to transect the mass with approximately 1-cm gastric margins. There were a few enlarged lymph nodes in the area and two biopsy specimens from the gastrohepatic ligament lymph nodes were taken for permanent sectioning.

**Figure 3:**
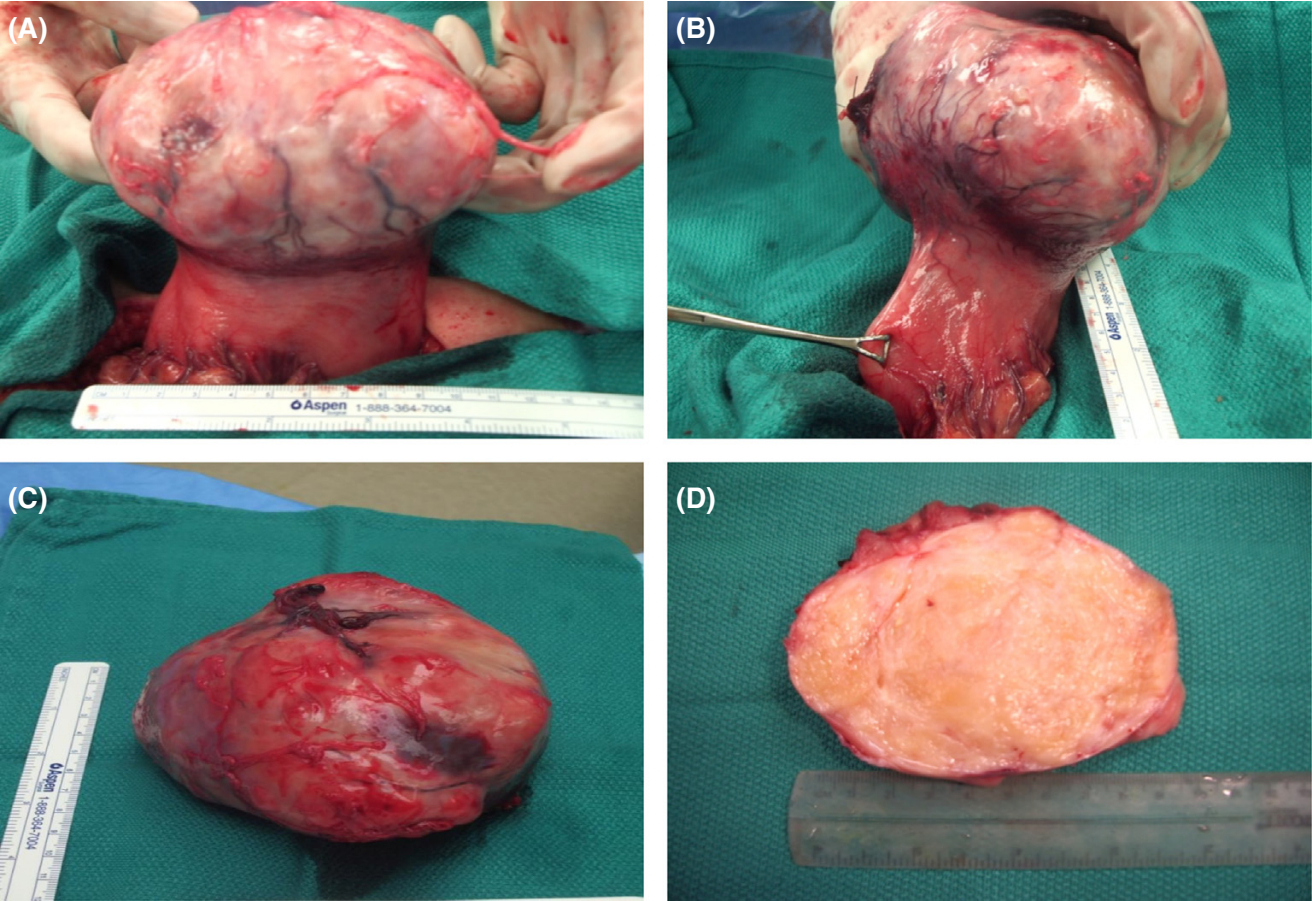
Specimen examination of the ovoid mass. No mucosal penetration was identified, and the mucosa slid freely over the lesion.

**Figure 4:**
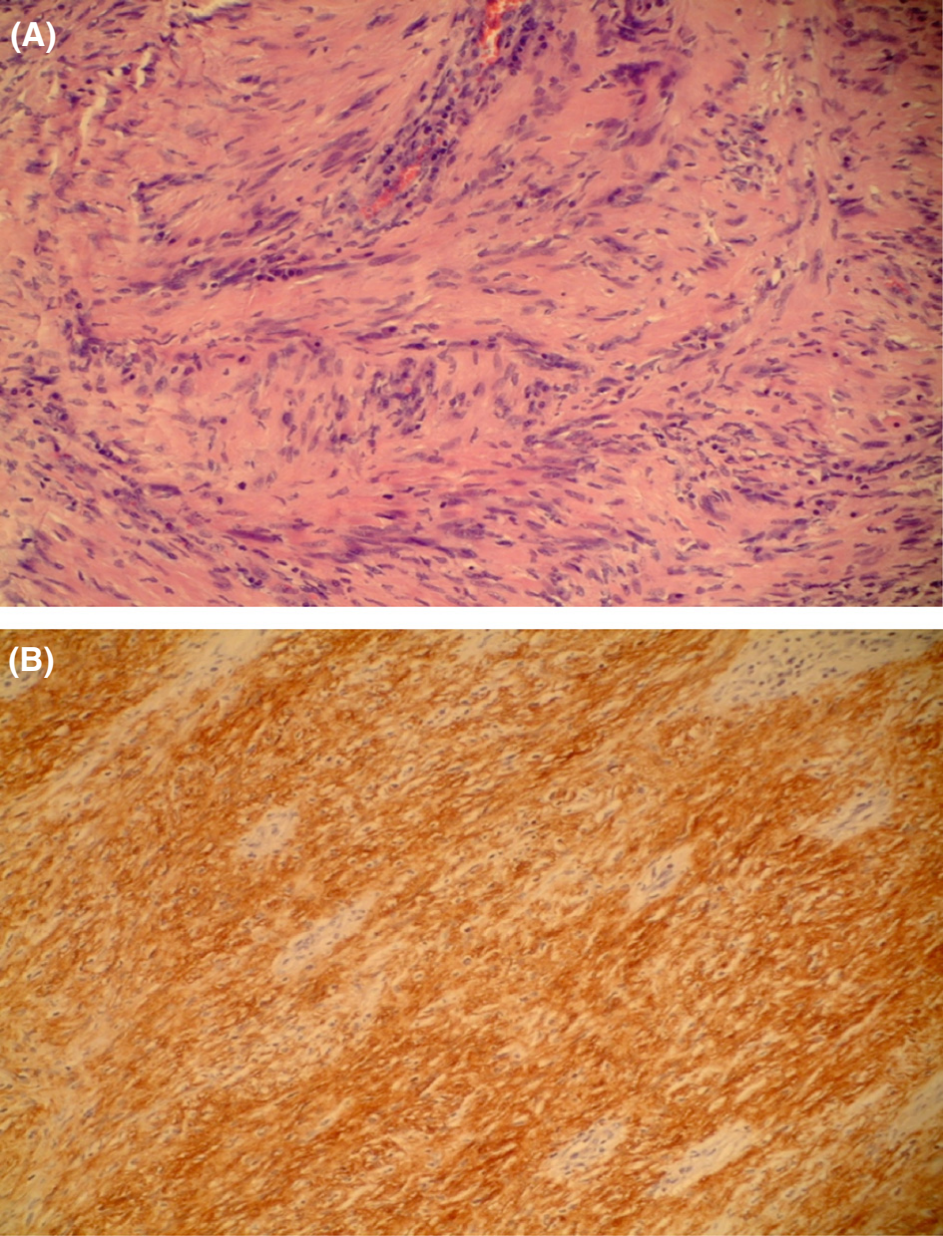
Histological and immunohistochemical features were consistent with a peripheral nerve sheath tumor or a benign schwannoma supporting the initial needle biopsy.

Specimen examination revealed an 11.2 × 8.9 × 8.0 cm ovoid mass with an attached 4.2 × 3.8 cm area of tan mucosa with somewhat flattened folds (Fig.3A–C). No mucosal penetration was identified, and the mucosa slid freely over the lesion. Submucosal mass sectioning revealed a white to yellow, gelatinous, firm, somewhat trabeculated cut surface with pinkish areas (Fig.3D). No necrosis or hemorrhage was identified. A thin, fibromembranous capsule surrounding the mass was intact. The gastric mucosa showed signs of chronic inflammation and no evidence of malignancy. The two lymph nodes showed signs of reactive lymphoid hyperplasia. Histology and immunohistochemistry revealed positive S100 and vimentin staining, and negative keratin, smooth muscle actin, CD34, and CD117 (Fig.4A and B). These data were indicative of peripheral nerve sheath tumor or a benign schwannoma supporting the initial needle biopsy.

## Discussion

Schwannomas (neurinomas/neurilemmomas) can originate in any nerve that has a Schwann cell sheath. They are generally benign and slow growing and develop more commonly in the stomach. GS are a rare, usually solitary, Gl mesenchymal tumor (0.2% of gastric tumors and 4% of benign gastric neoplasms) 1,6–12. Immunohistochemical or ultrastructural results are used to distinguish them from other gastric mesenchymal tumors 8,9. With the aid of immunohistochemical staining, Sarlom o-Rikala et al. 10 and Christopher et al. 2 reported the differences between gastric spindle-cell tumors. GS commonly occurs in female patients aged 50–60 years 6,10. Often times they are discovered incidentally at laparotomy or radiography and normally are asymptomatic, but some patients may have abdominal pain or discomfort, or even occult upper GI bleeding from ulceration due to emerging submucosal mass, constricting blood supply to the gastric mucosa 2,13.

When patients suffer from upper GI bleeding, evaluation is usually by endoscopic examination 7. For patients with a gastric mass, chest radiography should be obtained to detect extra-gastric pulmonary lesions. Occasionally, gastric tumors can be found during chest radiography; although, this would not be the initial imaging modality of choice. Upper GI series with barium contrast is a useful tool to localize the lesion and understand relationships of the mass to the esophagus and stomach. The extent of invasion and the type of lesion can be determined by CT scan. On CT they appear to be well-defined submucosal mass-like lesions with diffuse enhancement 7. Sonography usually depicts a homogenously hypoechoic mass 13. On MRI examination, T1-weighted images show low overall signal pattern and T2 images show moderate to markedly elevated pattern. For diagnostic purposes, the current standard of care and most accepted route of entry for a biopsy prior to surgery is endoscopically (as for gastric tumors).

The use of percutaneous biopsy for potentially neoplastic lesions has been controversial. A long-held theory is that there is a possibility of tumor seeding along the biopsy path, as well as a risk of tumor rupture into the abdomen and spreading of tumor cells through peritoneal or mesenteric seeding. Contrary to popular belief, recent evidence suggests this consequence is highly unlikely. In a review by Marco-Domenech et al. 3 only four cases of such complications were described. These authors and others concluded that not only was the procedure far better tolerated than the endoscopic route, but that a percutaneous US-guided biopsy can be safely and accurately obtained with minimal side effects or long-term sequelae 3,4.

Based on current literature, the best treatment option for GC is complete surgical resection (subtotal resection, near-total resection, or wedge resection). Surgical resection is usually curative with prompt relief of symptoms.

Percutaneous biopsy is better tolerated than the endoscopic route, safe and accurate, and can be obtained via a small gauge US-guided percutaneous needle with minimal side effects or long-term sequelae.

## Conflict of Interest

None declared.
